# The Earliest Matches

**DOI:** 10.1371/journal.pone.0042213

**Published:** 2012-08-01

**Authors:** Naama Goren-Inbar, Michael Freikman, Yosef Garfinkel, Nigel A. Goring-Morris, Leore Grosman

**Affiliations:** Institute of Archaeology, The Hebrew University of Jerusalem, Mt. Scopus, Jerusalem, Israel; University of Florence, Italy

## Abstract

Cylindrical objects made usually of fired clay but sometimes of stone were found at the Yarmukian Pottery Neolithic sites of Sha‘ar HaGolan and Munhata (first half of the 8^th^ millennium BP) in the Jordan Valley. Similar objects have been reported from other Near Eastern Pottery Neolithic sites. Most scholars have interpreted them as cultic objects in the shape of phalli, while others have referred to them in more general terms as “clay pestles,” “clay rods,” and “cylindrical clay objects.” Re-examination of these artifacts leads us to present a new interpretation of their function and to suggest a reconstruction of their technology and mode of use. We suggest that these objects were components of fire drills and consider them the earliest evidence of a complex technology of fire ignition, which incorporates the cylindrical objects in the role of matches.

## Introduction

Elongated cylindrical objects were first found in the Near East at the Pottery Neolithic (PN, 8^th^ millennium BP) site of Sha‘ar Hagolan by Stekelis [1] and later at the PN site of Telulyot Batashi by Kaplan [2]. These objects were deemed phalli on the basis of their shape and interpreted as cultic in purpose [3]. At the PN site of Munhata, 16 similar clay items [4] and four stone items [5] were reported. In a preliminary report such items were illustrated together with anthropomorphic figurines [6], and in the publication of the stone items they were described as “schematic and naturalistic representations of phalli” [5].

**Figure 1:**
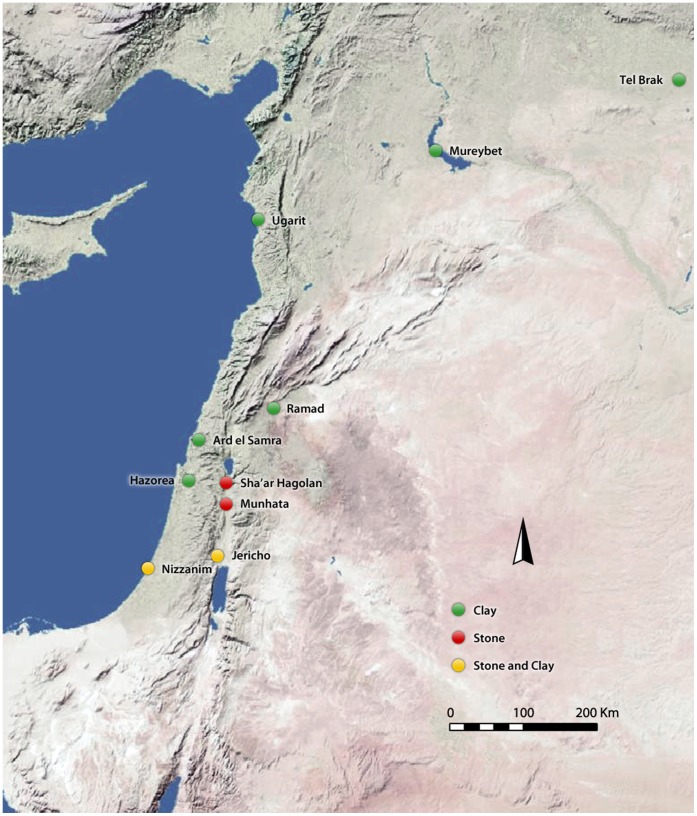
Distribution map of Neolithic sites mentioned in the text where cylindrical artifacts (both fired clay and stone) were found.

The largest assemblage of these items, some 80 complete and fragmentary objects that are all made of fired clay, was recovered during recent excavations at the PN site of Sha‘ar HaGolan [7], [8]. These objects are all cylindrical in shape and many have one conical end, though they are rarely biconical. Only ca. 19% of them are complete (n = 15). The assemblage at Sha‘ar HaGolan is assigned to the Yarmukian culture. Similar artifacts are known from several sites in northern Israel that are dated to the latter part of the 8^th^ millennium BP and the earlier part of the 7^th^ millennium BP [9]–[11] (note that Gopher [9] assigns the Yarmukian culture to the second rather than the first half of the 8^th^ millennium BP). The geographical distribution of these cylindrical artifacts (both stone and clay) is quite extensive and they have been reported from several other sites in the Near East (Figure 1). Stekelis, the first to discuss this category of object, suggested that they were cultic in function, following their presumed resemblance to phalli [1], [3]. Others have referred to them in a more generalized fashion as “clay pestles” [7], “clay rods” [12], or “cylindrical clay objects” [8].

We present here some new observations on the Sha‘ar HaGolan assemblage and discuss the characteristics of the cylindrical artifacts. We then reconsider the data and the phenomenon in general and suggest a new interpretation for the production, technology, and function of these artifacts: that they are matches – the tool used to ignite fire. In order to test this hypothesis, the clay cylinders were re-examined and compared with data stemming from archaeological, ethnographic, and experimental studies.

## Results

### Characterizing the Cylindrical Artifacts

**Figure 2:**
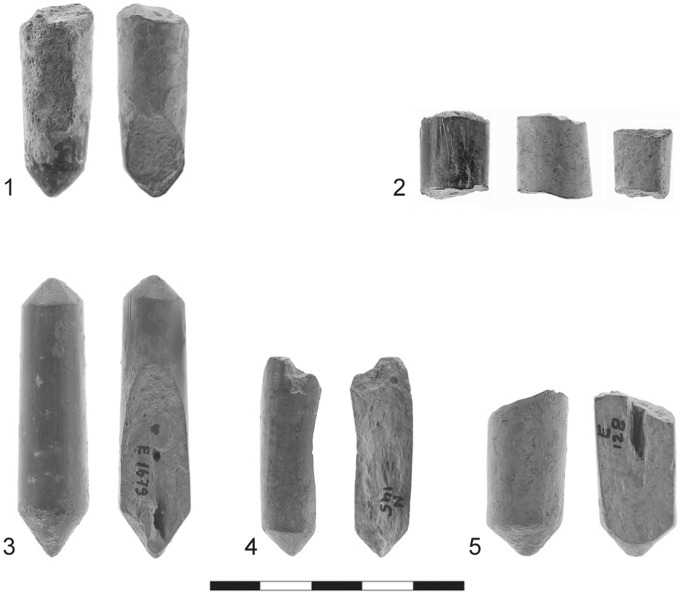
Fired-clay cylindrical artifacts. 1) darkened tip and typical longitudinal break; 2) medial breaks of three different artifacts; 3–5) three examples of artifacts with typical longitudinal break; the left-hand artifact is an example of the biconical type.

**Figure 3:**
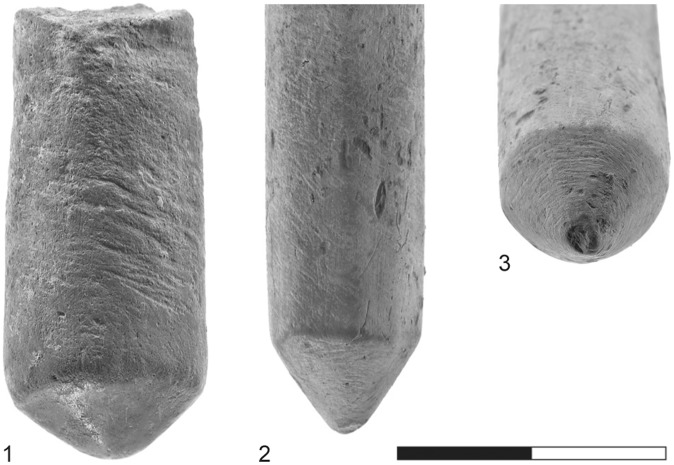
Characteristic traits of fired-clay cylindrical artifacts. 1) grooves; 2) grooves and striations; 3) darkened conical tip with associated striations resulting from rotation.

**Figure 4:**
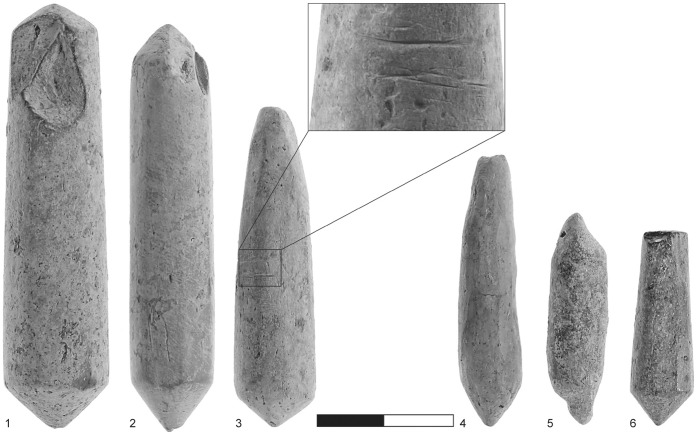
Fired-clay cylindrical artifacts. 1–2) biconical; 3, 4, 6) single conical tip; 3) usage grooves (mid-section and its enlargement); 5) pinched at both ends; 6) conical and flat ends.

The cylindrical objects are all made of high-quality, extremely fine-grained clay, with some grits less than 1 mm in size. The objects were fired at a relatively high temperature and their color varies between different shades of gray [8]. In general, the cylinders differ greatly in both fabric and color from the pottery assemblage recovered at Sha‘ar HaGolan; only a large human statue was made of this kind of clay [13]. The length of the complete items is 30–60 mm (mean: 46.9 mm) and the diameter is 12.6–14.1 mm. The cylindrical objects were produced by rolling the clay on hard, flat surfaces that left impressions of various types on their bodies. Although only 18.7% are complete [8], the morphology of the extremities (both complete and broken) is quite varied: flat, conical, double conical, rounded, pinched, rounded and conical, or unidentified (Figures 2, 3, 4).

The morphology of the cylinder is even and symmetrical along the entire length axis. Previous observations [8] yielded additional traits, including combing (20%), scraping (30%), and polishing (12.5%) or burnishing (26.3%). Summing up these characteristics, it is evident that some 71% of the entire assemblage bears signs of secondary treatment. This is especially striking in view of the efforts invested in the pottery vessels and figurines found on site [8].

Recent re-examination of the artifacts resulted in the identification of several additional features on some of the objects:

- Striations: Of major importance are the striations visible on the conical ends of the items (Figure 3∶3). Striations sometimes appear on the body of the item as well (Figure 3∶1).
- Dark coloration: Excessive heat has resulted in black staining (Figures 2∶1, 3∶3). This staining is in marked contrast to the grayish color of the cylindrical body and occurs only on the conical tips of artifacts. This coloration was apparently produced unintentionally and after the clay cylinders had been fired.
- A particular breakage pattern: Many of these objects are broken, displaying different breakage patterns. Some breaks are transversal (up to 30°; n = 42; 62.7% of the objects) (Figure 2∶2), others are oblique (45°; n = 31; 46.3%) (Figure 2∶5) or perpendicular to the length axis (up to 30°; n = 22; 27.5%) (Figure 2∶3–5). Altogether, 71.6% of breaks are oblique and perpendicular, clearly a deviation from the transversal breakage mode predicted for objects with elongated morphology.
- Grooves, deeper than the striations on the tips, are observed on the bodies of clay cylinders. There are thin grooves of roughly horizontal orientation (Figure 4∶3), while Figure 3∶1–2 illustrates shallower and more numerous oblique grooves, covering more of the surface.

Near Eastern evidence of fire production is extensive and begins as early as the Acheulian of Gesher Benot Ya‘aqov [14]. Throughout the Paleolithic era, indications for fire exploitation are provided by ashes [15]–[17], charcoal [19], and hearths [17]–[21], joined in the Epi-Paleolithic by remains of lime plaster [22], [23]. An extensive role of fire becomes more visible during the Pre-Pottery Neolithic A (PPNA; ca. 11,750–10,500 calBP; all dates cited from [24]) and particularly in the Pre-Pottery Neolithic B (PPNB; ca. 10,500–8,400 calBP), though we lack evidence for the production of fire. Clearly, the Neolithic material culture manifests a distinct evolutionary phase in pyrotechnology [25] as expressed by an unprecedented array of technologies associated with fire. Among these, a component of great importance is the introduction and the extensive use of “mechanical” drills.

Drilling has been documented as early as the Natufian culture (15,000–11,700 years calBP) through increased numbers of cap stones and drilled stones including beads [26]–[27]. Still, the evidence for the use of drills rises dramatically at the beginning of the Neolithic period (PPNA), as observed at the quarry sites of Hatula [28], Tzur Nathan [29], and Kaizer. Data are available from the sites of Netiv Hagdud [30] and Gilgal [31]–[33] as well. These manifestations, as well as those observed in the following PPNB cultures, include stone-ground vessels and implements such as basins, bowls, perforated rocks, and slabs; jewelry such as beads, pendants, and amulets [34]–[35]; bone tools such as points, fish hooks, needles, and buckles [32]; figurines of stone, clay, bone, and ivory; and even human bones [36]. Clearly, drilling technology was implemented for a diverse range of tasks and materials prior to the Pottery Neolithic cultures.

## Discussion

### The New Interpretation

Our interpretation is based on the known cultural and technological evidence for advanced pyrotechnology in Neolithic times. The various characteristics of the clay cylindrical objects described above (shape, symmetry, excellent clay quality, conical extremities, particular breakage patterns, spiral striations, and darkened tips in some case), can all be explained by a single function. We propose that these items are the earliest recorded matches – drill bits serving as a component of an advanced composite drill mechanism to produce fire. The basic property of this mechanism, well attested ethnographically [37], [38], is high-speed rotation to create friction. The rotation transmits energy, i.e., heat, in the socket of a fireboard, causing three types of markings through friction between the drill bit and the board. These are striations (including parallel striations, rotational marks, spiral scratches, and spiral grooves), polish marks, and darkening of the drill bit when extensive heat is generated. The heat generated by the friction ignites the tinder that is placed on the board, frequently in a groove or fire pan, a shallow depression containing the tinder (e.g., [37], [38]). In addition, the rotational motion, which entails both pressure and speed, gives the ends of the cylindrical objects a conical shape while they are rotated inside the board’s socket, sometimes enlarging the latter. Ethnographic [37], [39]–[41] and experimental [38], [41] studies show that the motion of the fire drill results in an abrasive pattern on the drill, forming a conical shape at the tip of the drill bit. Some of the specimens were given a conical end in advance during the primary stage of modification, probably in order to ease the drill bit into the fireboard (Figure 4∶5).

**Figure 5:**
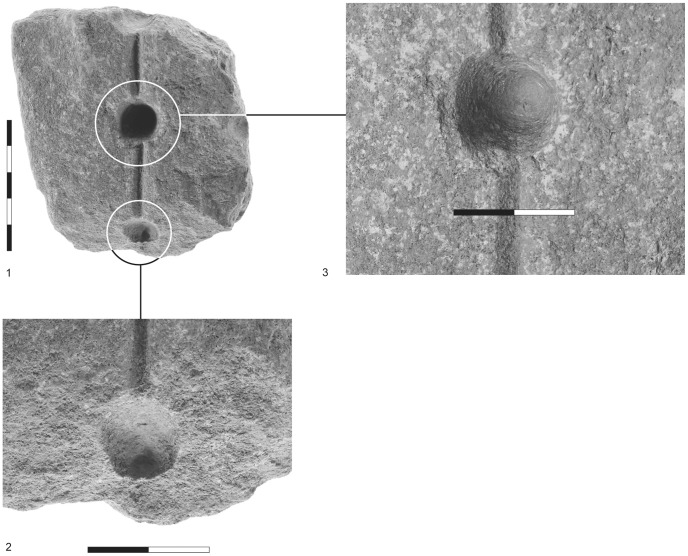
Kfar HaHoresh limestone artifacts interpreted as fire boards. 1) sockets and groove; 2–3); close-up of sockets with striations and fire pan.

The speed of rotation, and the abrasive force that it generates, clearly depend on two main factors: the drill bit (its morphology and raw material) and the fireboard (i.e., the “hearth”). Archaeological examples of drills (both palm and bow varieties) and boards made of wood have been discovered in the Old World, mainly in Egypt (e.g., [42], [43]) but in Europe as well [38]. The New World furnishes similar evidence, notably from South America ( [38], [41] and references therein). Similar findings, but with a much more extensive variability of raw materials, are recorded from the ethnographic data [39], [40], [43].

**Figure 6:**
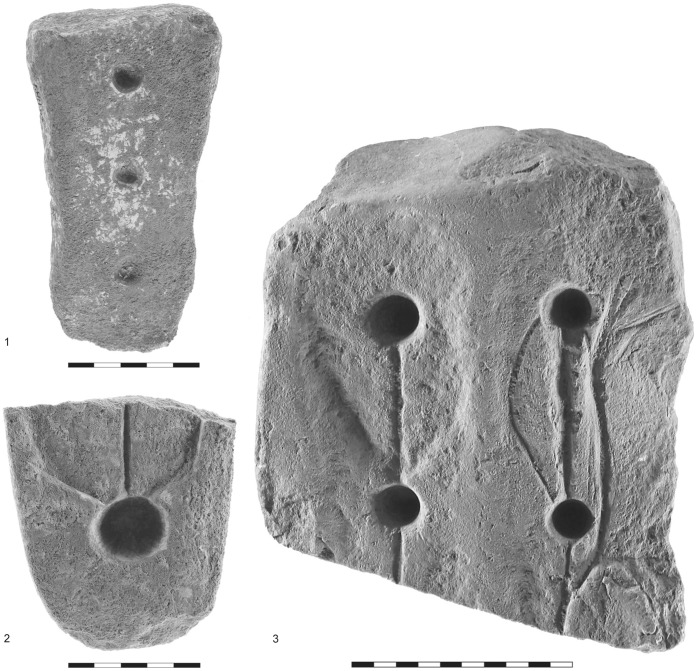
Kfar HaHoresh limestone artifacts interpreted as fire boards. 1) sockets; 2) socket, groove, and fire pan; 3) sockets, grooves, and fire pans.

Although reconstructing the drills used at Sha‘ar HaGolan is a speculative task, we propose two alternatives: a bow drill [40], [44]–[46] or a pump drill [37], [40], [44], [47]. These drill types comprise a drill bit, a shaft/spindle, a handle, a top piece, and cords/thongs. The pump drill has an additional component, a flywheel (whorl) usually in the form of a perforated thin disc, which serves as a weight to add momentum [37]. Both drill types require hafting devices and technologies to connect the drill bit to the shaft, and both use a board with sockets, usually lined up in a row, for the bit to drill into the surface of the board [39], [43]. Indeed, the Sha‘ar HaGolan clay assemblage includes objects [8], [48] that could easily have served as flywheels and fore-shafts to connect the drill bit to the rotating shaft.

Of the two varieties of drill, the bow drill seems the more appropriate, considering the presence of distinct damage pattern in the form of grooves (Figure 4∶3). These grooves probably resulted from the thin string of the bow rubbing the body of the clay cylinder. If this is a valid reconstruction, these grooves may be indicative of a very short drill bit without a shaft and with a capstone and a string wrapped around the cylindrical artifact.

The use of the cylindrical artifacts as drill bits to ignite fire is further supported by their different breakage patterns. Activating a pump/bow drill introduces pressure along the axis of the shaft/drill bit. With the bow drill this force is introduced from the top of the shaft (by hand/cap stone) and interacts with the rotational force caused by the movement of the bow. In the case of a pump drill, the friction in the board’s socket is produced mainly by the weight of the flywheel and to a lesser extent by the pressure of the hand on the handle [37]. In both instances transversal breakage may occur when equilibrium is lacking between the perpendicular vectors of force described above. In both drills any excessive force will cause breakage, and one may cite the high frequency of longitudinal and diagonal breaks at Sha‘ar HaGolan (Figures 2, 3).

The biconical cylinders exemplify the technological sophistication and flexibility of the Sha‘ar Hagolan inhabitants. The biconical artifacts are viewed as double-ended objects in which an exhausted end had been replaced, after rehafting, by the other end of the same clay cylinder. The items with one conical end and those with rounded ends could also have been reused after rehafting. The artifacts with pinched ends (Figure 4∶5) may be viewed as prepared but as yet unused items. Experimental work [38], [41] shows that the very first turns of the drill are difficult, as the drill bit is liable to slip from its intended point on the board. Thus, pinching can be a stabilizing factor maintaining the drill bit at a particular point on the board. According to ethnographic observations, the grip of the drill bit on the board can be improved by grooving/notching (or alternatively making a cross) at the drilling point on the board, but the grooves or notches are frequently also guttered in order to allow the accumulation of sawdust (e.g., [43]). The dimensions of the sockets increase with the ongoing and repeated process of drilling.

Based on the frequency of these clay objects and their lack of any artistic decoration, as well as the absence of any coloration (apart from the dark staining on the conical tips are related to charring), we conclude that these fired-clay objects were indeed drill bits employed in the process of fire making.

### The Origin and Spread of the Fire Drill

Archaeological evidence of fire drills is extremely rare, probably due to the fact that they were generally made of perishable materials, particularly wood. Both drill and board are preserved only under very particular conditions, either in hyper-arid or in waterlogged environments (e.g., the Egyptian wooden specimens mentioned above). In support of our interpretation we may refer to the Egyptian hieroglyph for fire, which portrays a fire drill of bow drill type [43]. Fire drills must have been a very common artifact type in antiquity to become an illustrative reference to fire.

As noted above, cylindrical artifacts have been found in a number of Near Eastern PN sites, including Munhata: clay [6], [49] and stone [11]; Jericho: stone [50]; Nizzanim: stone [51]; Ard el Samra: clay [52]; Ugarit: clay [53]; Mureybet: stone [54]; Tel Brak clay [55]; and Jfrabad: clay [56] (Figure 1). This widespread distribution illustrates the regular use of this particular method of igniting fire and the fact that it was a very common procedure during the PN.

Fired-clay cylindrical objects older than the PN (e.g., [33]) have not yet been examined in the light of the above interpretation; thus, it is possible that similar items do exist in the archaeological record preceding that of the PN and that their distribution is even wider. The PPNB site of Kfar HaHoresh furnishes evidence for possible use of fire drills prior to the PN. The evidence comprises several stone blocks made of limestone and assigned to the Middle PPNB. These fragmentary stone artifacts, two reported in [57], have one or more pits/sockets with grooves connecting them (Figures 4, 5, 6). Examination of the sockets and their morphology, as well as the straight and curved incisions on the stone block, leads us to consider these artifacts as fireboards, similar to objects recorded through ethnographic observations (e.g., [39]). Some of the Kfar HaHoresh artifacts also exhibit a shallow depressed surface around the perimeter of the sockets, which includes the incisions/grooves leading to the sockets (Figure 6). In our interpretation, the sockets were formed by the insertion of the drill bit and its rotation, which in turn enlarged the sockets as drilling advanced. The surface grooves were made to accommodate the tinder and the depression around it, known from ethnographic items, was hollowed in order to lay down additional tinder (usually as a heap or bundle) to prevent the wood dust from rising up from the spiral motion of the drilling. This is the fire pan, i.e., the area where tinder is laid on the board, to catch the spark and complete the process of ignition [37]. Apparently it is essential to keep the heated dust in a heap [39] and the fire pan clearly helps to sustain optimal conditions. Thus, we consider these (stone) artifacts the earliest manifestations of fire boards associated with the production of fire. Petrie wrote: “*Both the fire drill and bow drilling probably originated from the use of the bow and arrow*” [42∶59] and Francis shared the view that the bow drill [37∶61] “… *is evidently a variety of the ordinary bow and arrow, modified for drilling*.” Kfar HaHoresh, like most PPNB sites, is rich in arrowheads that imply knowledge of bow mechanics, further strengthening our suggestion that the systematic production of matches predates the PN finds at Sha‘ar HaGolan.

Archaeological and ethnographic descriptions of fire drills are concerned with wooden apparatus and with friction between two wooden elements. Nevertheless, we believe that the items presented here, though made of clay and stone, were used for the same purpose. The mechanism that produces heat and combustion is rooted in the friction of two elements in motion. Ethnographic data (e.g., [39]) indicate that in order to increase the friction, sand (or grit of other types) was poured into the socket. We lack the boards that were the counterpart of the fired-clay cylindrical artifacts, but the striations on the conical parts, and sometimes on other parts of the item, demonstrate that extensive friction did take place. The holed stone boards from Kfar HaHoresh provide evidence that the friction was intense. The Pre-Pottery Neolithic artisans were skilled in drilling stone and other hard materials (e.g., [28] and see references above). The wide geographical distribution of the fired-clay and stone cylinders emphasizes this point. The increasingly frequent occurrence of partially perforated stone blocks described as “game boards” at other Near Eastern PPNB sites, such as Beidha [58], Wadi Tbeik [59], ‘Ain Ghazal [60], Wadi Abu Tulayaha [61]–[64] and Wadi Ghwair [65], clearly merits further investigation. Of these, some of these could have functioned as fireboards.

### The Symbolic Aspect

Our interpretation of these items does not negate the symbolic connotations that they may have held, as pointed out by Stekelis [3]. Ethnographically, in many societies the fire drill and the fireboard are considered to represent the male and female sex organs respectively. One can thus add this aspect to the importance of fire drills, probably first introduced in the Pre-Pottery Neolithic and becoming common in the Pottery Neolithic period.

## Materials and Methods

Cylindrical clay artifacts originating in the excavations of the Pottery Neolithic site of Sha‘ar HaGolan provide the material for the analysis [3], [4], [7], [8], [12]. Attribute analyses of morphology, damage marks (breakage, striations, grooves), and color are described, and some examples are illustrated. The interpretation of the clay objects relies on experimental data [37], [38], [41] and ethnographic studies [39], [40], [43], [44], [46], [47], as well as complete sets of archaeological fire drills that are assigned to later periods [42], [43], [45].

## References

[1] Stekelis M (1951) A new Neolithic industry: The Yarmukian of Palestine. IEJ 1: 1–19.

[2] Kaplan J (1958) Excavations at Teluliot Batashi, Nahal Soreq. Eretz Israel 5: 9–24.

[3] Stekelis M (1972) The Yarmoukian Culture of the Neolithic period. Jerusalem: Magness Press. p. 45.

[4] Garfinkel Y (1995) Human and animal figurines of Munhata (Israel). Paris: Association Paléorient. p. 127.

[5] Gopher A, Orrelle E (1995) The groundstone assemblages of Munhata. A Neolithic site in the Jordan Valley, Israel: a report. Paris: Association Paléorient. p. 168.

[6] Perrot J (1964) Les deux premières campagnes de fouilles à Munhata (1962–63), premiers résultats. Syria 41: 323–345.

[7] Garfinkel Y (1993) The Yarmukian Culture in Israel. Paléorient 19(1): 115–133.

[8] Freikman M (2006) The Assemblage of baked clay items of Sha‘ar Hagolan: a Pottery Neolithic site in the Jordan Valley. Jerusalem: The Hebrew University MA dissertation. p. 137.

[9] Garfinkel Y, Ben Shlomo D (2009) Sha‘ar Hagolan Vol. 2: The rise of urban concepts in the ancient Near East. Jerusalem: Qedem Reports. 308.

[10] Gopher A (1993) Sixth–fifth millennia B.C. settlements in the Coastal Plain in Israel. Paléorient 19(1): 55–64.

[11] Gopher A, Orrelle E (1996) An alternative interpretation for the material imagery of the Yarmukian, a Neolithic culture of the sixth millennium BC in the southern Levant. Cam Archaeol J 6(2): 255–279.

[12] Garfinkel Y, Miller MA (2002) Sha‘ar Hagolan Vol. 1. Neolithic art in context. Oxford: Oxbow Books. p. 262.

[13] Garfinkel Y, Ben-Shlomo D, Korn N (2010) Symbolic dimensions of the Yarmukian culture: canonization in Neolithic art. Jerusalem: Israel Exploration Society Press. p. 353.

[14] Alperson-Afil N, Goren-Inbar N (2010) The Acheulian site of Gesher Benot Ya‘aqov Vol. II: ancient flames and controlled use of fire. Dordrecht: Springer. p. 120.

[15] Schiegl S, Goldberg P, Bar-Yosef O, Weiner S (1996) Ash deposits in Hayonim and Kebara Caves, Israel: Macroscopic, microscopic and mineralogical observations, and their archaeological implications. J Archaeol Sci 23: 763–781.

[16] Karkanas P, Shahack-Gross R, Ayalon A, Bar-Matthews M, Barkai R, et al (2007) Evidence for habitual use of fire at the end of the Lower Paleolithic: Site-formation processes at Qesem Cave, Israel. J Hum Evol 53(2): 197–212. 17572475 10.1016/j.jhevol.2007.04.002

[17] Valla FR, Khalaily H, Samouelian N, March R, Bouquentin F, et al (2001) Le Natoufien Final de Mallaha (Eynan), deuxième rapport préliminaire: Les Fouilles de 1998 et 1999. J Israel Preh Society 31: 43–184.

[18] Schick T, Stekelis M (1977) Mousterian assemblages in Kebara Cave, Mount Carmel. Eretz Israel 13: 97–149.

[19] Goring-Morris AN (1987) At the edge: Terminal Pleistocene hunter-gatherers in the Negev and Sinai. Oxford: BAR International. p. 526.

[20] Bar-Yosef O, Belfer-Cohen A, Goldberg P, Kuhn S, Meignen L, et al (2005) Archaeological background to Hayonim Cave and Meged Rockshelter. In: Stiner MC, editor. The faunas of Hayonim Cave, Israel. (Peabody Museum, Harvard University, Cambridge) 17–38..

[21] March RJ. Searching for fire structures function and formation process: a geochemistry approach. In: Bar-Yosef O, Valla FR, editors. The Natufian culture of the Levant II. Ann Arbor: International Monographs in Prehistory. In Press..

[22] Kingery DW, Vandiver PB, Pickett M (1988) The beginnings of pyrotechnology, part II: Production and use of lime and gypsum plaster in the Pre-Pottery Neolithic Near East. J Field Archaeol 15: 219–244.

[23] Bar-Yosef O (1991) The Archaeology of the Natufian layer at Hayonim Cave. In: Bar-Yosef O, Valla FR, editors. The Natufian culture in the Levant. Ann Arbor: International Monographs in Prehistory. 81–92.

[24] Goring-Morris N, Belfer-Cohen A. The Southern Levantine Neolithic in and West of the Rift Valley. In: Steiner M, Killebrew AE, editors. The Oxford Handbook of the Archaeology of the Levant (ca. 8000–332 BCE). Oxford: Oxford University Press. In press..

[25] Goren Y, Goring-Morris AN (2008) Early Pyrotechnology in the Near East: Experimental Lime Plaster Production at the Pre-Pottery Neolithic B Site of Kfar HaHoresh, Israel. Geoarchaeol 23(6): 779–798.

[26] Edwards PC (1991) Wadi Hammeh 27: An Early Natufian site at Pella, Jordan. In: Bar-Yosef O, Valla FR, editors. The Natufian culture in the Levant. Ann Arbor: International Monographs in Prehistory. 123–148.

[27] Belfer-Cohen A (1991) The Natufian in the Levant. Annu Rev Anthropol 20: 167–186.

[28] Grosman L, Goren- Inbar N (2007) “Taming” Rocks and Changing Landscapes. A New Interpretation of Neolithic Cupmarks. Curr Anthropol 48(5): 732–740.

[29] Marder O, Goring-Morris AN, Khalaily H, Milebski I, Rabinovich R, et al (2007) Tzur Natan, a Pre-Pottery Neolithic A Site in Central Israel and Observations on Regional Settlement Patterns. Paléorient 33(2): 79–100.

[30] Gopher A (1997) Ground stone tools and other stone assemblages from Netiv Hagdud. In: Bar-Yosef O, Gopher A, editors. An Early Neolithic village in the Jordan Valley. Part 1: The Archaeology of Netiv Hagdud. Cambridge: Peabody Museum, Harvard University. 151–176.

[31] Noy T (1979) Stone Cup-Holes and Querns from Gilgal I, A Pre-Pottery Neolithic Site in Israel. Paléorient 5: 233–238.

[32] Belfer-Cohen A (2010) Bone tools from the Gilgal sites. In: Bar-Yosef O, Goring-Morris AN, Gopher A, editors. Gilgal - Early Neolithic occupations in the Lower Jordan Valley. The excavations of Tamar Noy. Oakville: ASPR & David Brown/Oxbow. 177–184.

[33] Hershman D, Belfer-Cohen A (2010) “It’s Magic!”: artistic and symbolic material manifestations from the Gilgal sites. In: Bar-Yosef O, Goring-Morris AN, Gopher A, editors. Gilgal - Early Neolithic occupations in the Lower Jordan Valley. The excavations of Tamar Noy. Oakville: ASPR & David Brown/Oxbow. 185–216.

[34] Garfinkel Y (1987) Bead manufacture on the Pre-Pottery Neolithic B site of Yiftah'el. J Israel Preh Society 20: 79–90.

[35] Bar-Yosef Mayer DE (2005) The Exploitation of Shells as Beads in the Palaeolithic and Neolithic of the Levant. Paléorient 31(1): 176–185.

[36] Simmons T, Goring-Morris AN, Horwitz LK (2007) “What ceremony else?” Taphonomy and the ritual treatment of the dead in the Pre-Pottery Neolithic B mortuary complex at Kfar Hahoresh, Israel. In: Faerman M, Horwitz LK, Kahana T, Zilberman U, editors. Faces from the Past: Diachronic patterns in the biology and health status of human populations from the Eastern Mediterranean. Oxford: Archaeopress, BAR International. 1–27.

[37] Francis PH (1961) The origin and developments of fire arms and gunpowder. Bradford: Broadacre Books. p. 124.

[38] Collina-Girard J (1993) Feu par percussion, feu par friction - les données de l’expérimentation. BSF 90(2): 159–173.

[39] Hough W (1888) Fire-Making Apparatus in the U. S. National Museum. Proceeding U.S. National Museum, Vol. 73, Washington. 531–587.

[40] Hough W (1890) Aboriginal fire-making. Amer Anthropol 3(4): 359–372.

[41] Collina-Girard J (1998) Le feu avant les allumettes: expérimentation et mythes techniques. Paris: Éditions de la maison des sciences de l’homme. p. 146.

[42] Petrie WMF (1917) Tools and weapons. London: British School of Archaeology in Egypt. p. 71.

[43] Harrison HS (1954) Fire-making, fuel and lighting. In: Singer C, Holmyard EJ, Hall AR, editors. A History of Technology. Oxford: Clarendon. 216–237.

[44] Davidson DS (1947) Fire-making in Australia. Amer Anthropol 49(3): 426–437.

[45] Petrie WMF (1884) On the mechanical methods of the ancient Egyptians. J Anthropol Inst 13: 88–109.

[46] Martin PS (1934) The bow-drill in North America. Amer Anthropol 36(1): 94–97.

[47] McGuire JD (1892) Materials, apparatus and processes of the aboriginal lapidary. Amer Anthropol A5(2): 165–176.

[48] Garfinkel Y (1999) The Yarmukians: Neolithic art from Sha‘ar Hagolan. Jerusalem Bible Land Museum. p. 96.

[49] Garfinkel Y (1992) The pottery assemblages of the Sha‘ar Hagolan and Rabah Stages of Munhata (Israel). Paris: Association Paléorient. p. 360.

[50] Dorrell PG (1983) Appendix A: Stone vessels, tools and objects, Jericho V. In: Kenyon K, Holland TA, editors. London: British School of Archaeology in Jerusalem. 485–576.

[51] Yeivin E, Olami Y (1979) Nizzanim - A Neolithic site in Nahal Evtah: Excavations of 1968–1970. Tel Aviv 6: 99–135.

[52] Getzov N, Barzilai O, Dosseur GI, Eirikh-Rose A, Ktalav I, et al (2009) Nahal Betzet II and Ard el Samra: Two Late Prehistoric sites and settlement patterns in the Akko Plain. J Israel Preh Society 39: 81–158.

[53] de Contenson H (1992) Préhistoire de Ras Shamra. Paris: ERC. p. 283.

[54] Cauvin J (1977) Les fouilles de Mureybet (1971–1974) et leur signification pour les origines de la sédentarisation au Proche-Orient. AASOR 44: 19–48.

[55] Matthews R (2002) editor (2002) Exploring an Upper Mesopotamian regional centre 1994–1996. Cambridge: McDonald Institute for Archaeological Research & British School of Archaeology in Iraq. p. 446.

[56] Dollfus G (1972) Les fouilles a Djaffarabad de 1969 a 1971. CDFAI 1: 17–75.

[57] Goring-Morris AN (2005 ) Kefar HaHoresh. In: Stern E, editor. The new encyclopedia of archaeological excavations in the Holy Land. Jerusalem: Israel Exploration Society and Simon & Schuster. 1907–1909.

[58] Kirkbride D (1966) Five seasons at the Pre-Pottery Neolithic village of Beidha in Jordan. PEQ 98: 8–72.

[59] Bar-Yosef O (1980) The Palaeolithic of Sinai. In: Meshel Z, Finkelstein I, editors. Qadmoniot Sinai: Sinai in antiquity. Tel Aviv Hakkibutz Hameuchad. 11–40.

[60] Rollefson G (1992) A Neolithic game board from ‘Ain Ghazal, Jordan. BASOR 286: 1–5.

[61] Fujii S (2006) Wadi Abu Tulayha: A Preliminary report on the 2005 Spring and summer excavation Seasons of the al-Jafr Basin prehistoric project, phase 2. ADAJ 50: 9–31.

[62] Fujii S (2007) Wadi Abu Tulayaha: A preliminary report on the 2006 summer fields Season of the Jafr Basin prehistoric project, phase 2. ADAJ 51: 373–402.

[63] Fujii S (2008) Wadi Abu Tulayaha: A preliminary report of the 2007 summer field Season of the Jafr Basin prehistoric project, phase 2. ADAJ 52: 446–478.

[64] Fujii S (2009) Wadi Abu Tulayha: A preliminary report on the summer 2008 final field season of the Jafr Basin prehistoric project, phase 2. ADAJ 53: 173–209.

[65] Simmons AH, Najjar M (2006) Ghwair I: A Small, Complex Neolithic Community in Southern Jordan. J Field Archaeol 31(1): 77–95.

