# Correction: The Earliest Matches

**DOI:** 10.1371/annotation/b52d7ab3-3099-49e1-b4b5-30b76cf153b5

**Published:** 2012-08-09

**Authors:** Naama Goren-Inbar, Michael Freikman, Yosef Garfinkel, A. Nigel Goring-Morris, Leore Grosman

There was an error in the fourth author's name. A. Nigel Goring-Morris is correct. The correct citation is: 

Goren-Inbar N, Freikman M, Garfinkel Y, Goring-Morris AN, Grosman L (2012) The Earliest Matches. PLoS ONE 7(8): e42213.

